# Supplementary material for: Biotic interactions promote local adaptation to soil in plants
Source: Nat Commun. 2024 Jun 18;15:5186. doi: 10.1038/s41467-024-49383-x (PMC11189560; doi:10.1038/s41467-024-49383-x)
Supplement: Supplementary file 1 — Supplementary Info [file 41467_2024_49383_MOESM1_ESM.pdf]

Biotic interactions promote local adaptation to soil in plants

Thomas Dorey[1,2], Léa Frachon[1,3], Loren H. Rieseberg[4], Julia Kreiner[4] and Florian P. Schiestl[1]*

[1] Department of Systematic and Evolutionary Botany, University of Zürich, Zürich, Switzerland

[2] present address: Department of Environmental Sciences, University of Basel, Basel, Switzerland

[3] present address: Agroécologie, INRAE, Institut Agro, Univ. Bourgogne, Univ. Bourgogne Franche-Comté, F-21000 Dijon, France

[4]Department of Botany and Biodiversity Research Centre, University of British Columbia, Vancouver, Canada

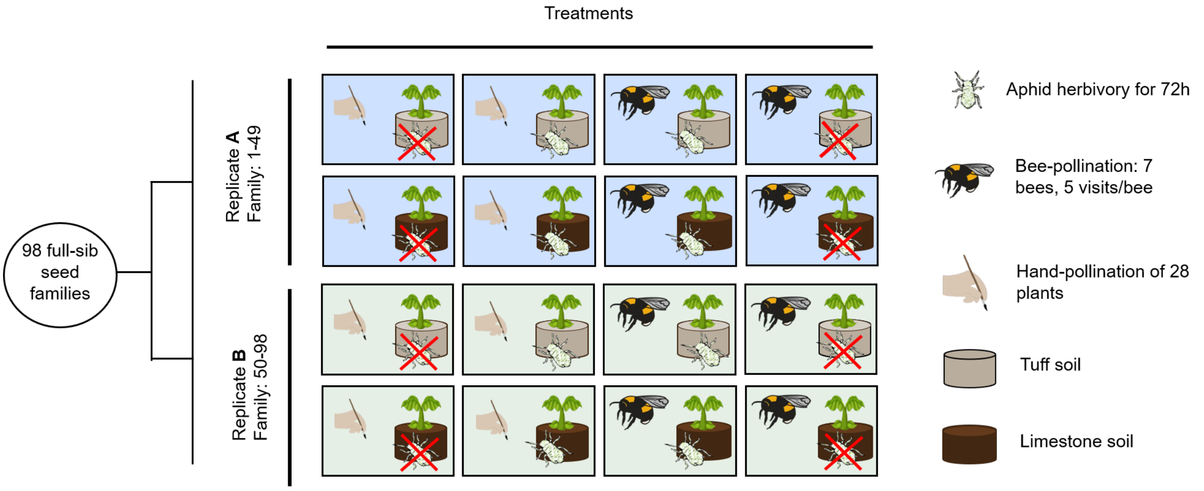

Supplementary Figure 1: **Design of our experimental evolution study.** 98 full-sib seed families of fast cycling *Brassica rapa* plants were divided into two replicates (A and B, 49 plants per replicate), shown in the blue- and green shaded squares, and spread among treatment groups, with each treatment containing all seed families. Soil: limestone (blue), tuff (green), herbivory (no aphids, aphids) and pollination (hand, bumblebee) were the factors in the experiment (*1*).

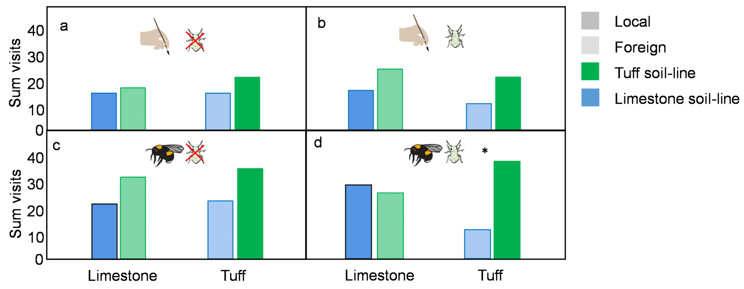

Supplementary Figure 2: Attractiveness of plants measured by first choices of bumble bees. a: in plants that evolved with hand pollination and no-herbivory (N=232), **b:** in plants that evolved with hand pollination and herbivory (N=224), **c:** in plants that evolved with bee-pollination and no-herbivory (N=232), **d:** that evolved with bee-pollination and herbivory (N=224). The graph shows plants that evolved in limestone (blue bars) and those that evolved in tuff soil (green bars) grown in either limestone- or tuff soil (as indicated on the x-axis label). In the treatment group bee-pollination and herbivory, local tuff-line plants were significantly more attractive than limestone-line plants when grown in tuff (* F_{104}=12.48, P=4.120*10^{-04}). Significance was determined using a two-sided generalized mixed with number of visits as dependent variable, soil and soil lines and their interaction as fixed factors and replicate as random factors.

Supplementary Table 1: Association between plant traits and relative seed set in plants with bee-pollination in generation one and two (combined, (*1*)), **and seven,** estimated by two-sided generalized linear model. Bold: P<0.05, italics: 0.05<P<0.10. Because of the zero-inflated distribution of fitness, fitness was treated as binary variable (seeds/no seeds) in A) and assessed only for plants that produced seeds (truncated model; relative seeds seed>0) in B). Plant traits were used as covariates and replicate as random factor in the models (na: traits were not available).

A)

	Generation 1 and 2 (N=684)				Generation seven (N=192)		
	df	Chsq	Odds ratio	P	Chsq	Odds ratio	P
**Number of open flowers**	**1**	**43.86**	**1.25**	**$3.533^{*}10^{-11}$**	**5.07**	**1.13**	**0.024**
Height at day 30 (cm)	1	0.33	0.99	0.565	*3.28*	*1.06*	*0.070*
**Time to first flower**	**1**	**10.12**	**1.13**	**0.001**	0.00	1.13	0.991
Flower diameter (cm)	1	2.66	0.82	0.116	0.49	1.13	0.483
Petal length (cm)	1	0.22	2.40	0.642	0.99	12.06	0.320
Petal width (cm)	1	1.92	23.44	0.166	0.07	2.33	0.797
Style length (cm)	1	0.55	0.42	0.459	0.02	1.30	0.883
Stamen length (cm)	1	1.67	12.59	0.197	**7.36**	**0.00**	**0.007**
Herkogamy	1	0.12	0.54	0.730	0.00	0.94	0.981
Nectar (log)	1	0.47	1.23	0.491	2.72	0.68	0.099
**Benzaldehyde (log)**	**1**	**4.34**	**1.52**	**0.038**	na	na	na
1- Butene, 4- isothiocyanate (log)	1	1.43	1.12	0.231	na	na	na
Methyl benzoate (log)	1	0.02	0.98	0.889	na	na	na
Phenylethyl alcohol (log)	1	2.70	0.75	0.100	na	na	na
2-Amino benzaldehyde (log)	1	0.95	0.90	0.330	na	na	na
p-Anisaldehyde (log)	*1*	*3.13*	*0.82*	*0.077*	na	na	na
Methyl anthranilate (log)	1	0.85	1.13	0.357	na	na	na
(*Z*)-3-Hexen-1-ol, acetate (log)	1	0.73	1.08	0.394	na	na	na
Phenylacetaldehyde (log)	1	0.05	1.03	0.817	na	na	na
Benzyl nitrile (log)	1	0.06	1.04	0.807	na	na	na
Methyl salicylate (log)	1	0.21	0.94	0.647	na	na	na
Indole (log)	1	2.67	1.20	0.102	na	na	na
(E.E)-α-Farnesene *(log)*	*1*	*3.14*	*0.80*	*0.076*	na	na	na
**Replicate**	**1**	**5.46**	**na**	**0.019**	0.44	na	0.505

B)

	Generation 1 and 2 (N=509)				Generation seven (N=93)			
	R^2	Chsq	β ± se	P	R^2	Chsq	β ± se	P
**Number of open flowers**	**0.10**	**46.68**	**0.14±0.06**	**0.015**	**0.23**	**9.46**	**0.19±0.06**	**0.002**
Height at day 30 (cm)	0.06	0.52	0.00±0.03	0.963	0.15	0.34	-0.02±0.04	0.561
Time to first flower	*0.06*	*9.61*	*0.12±0.07*	*0.096*	0.17	2.16	-0.08±0.06	0.141
Flower diameter (cm)	0.06	2.12	-0.01±0.22	0.953	0.14	0.29	-0.14±0.25	0.592
**Petal length (cm)**	0.06	0.06	-0.78±3.52	0.768	**0.14**	**5.42**	**11.32±4.86**	**0.020**
Petal width (cm)	0.06	1.68	1.14±3.86	0.824	0.14	0.13	-1.44±4.03	0.719
**Style length (cm)**	0.06	0.46	3.07±1.89	0.104	**0.17**	**10.36**	**-6.34±1.97**	**0.001**
Stamen length (cm)	0.07	1.01	1.11±3.49	0.749	0.14	0.04	-0.88±4.24	0.835
Herkogamy	0.06	0.92	-2.93±3.29	0.371	0.14	0.20	1.28±2.87	0.655

Nectar (ln)	0.06	0.41	-0.32±0.52	0.539	0.14	1.33	-0.27±0.24	0.249
Benzaldehyde (ln)	0.03	0.29	-0.33±0.38	0.374	na	na	na	na
1- Butene, 4- isothiocyanate (ln)	0.03	0.66	-0.02±0.17	0.919	na	na	na	na
Methyl benzoate (ln)	0.03	0.30	-0.28±0.25	0.244	na	na	na	na
Phenylethyl alcohol (ln)	0.03	1.87	-0.03±0.27	0.922	na	na	na	na
**2-Amino benzaldehyde (ln)**	**0.04**	**4.43**	**-0.60±0.22**	**0.006**	na	na	na	na
p-Anisaldehyde (ln)	0.03	0.08	0.14±0.19	0.463	na	na	na	na
Methyl anthranilate (ln)	0.03	0.92	0.37±0.24	0.118	na	na	na	na
(*Z*)-3-Hexen-1-ol, acetate (ln)	0.03	0.94	0.07±0.15	0.647	na	na	na	na
Phenylacetaldehyde (ln)	*0.03*	*1.83*	*0.36±0.21*	*0.088*	na	na	na	na
Benzyl nitrile (ln)	0.03	0.01	-0.15±0.31	0.620	na	na	na	na
Methyl salicylate (ln)	0.03	0.01	0.21±0.24	0.386	na	na	na	na
Indole (ln)	0.03	0.66	0.16±0.20	0.419	na	na	na	na
(E.E)-α-Farnesene (ln)	0.03	0.19	0.07±0.21	0.761	na	na	na	na
Replicate	na	0.61	na	0.714	na	0.02	na	0.888

Supplementary Table 2: Association between plant traits and bumblebee first choices in plants of generation ten with an evolutionary history of bee-pollination. Significances were determined using two-sided generalized linear model, bumblebee first choices were the dependent variable, traits covariates and replicate a random factor.

	Generation 10 (N=406)		
	df	Chsq	P
**Number of open flowers**	**1**	**5.36**	**0.021**
Height at day 30 (cm)	1	1.44	0.231
Leaf size (cm2)	1	2.05	0.152
Length per branch (cm)	1	0.09	0.768
Time to first flower	1	0.49	0.484
Flower diameter (cm)	1	0.25	0.620
Petal length (cm)	1	0.59	0.442
Petal width (cm)	1	1.34	0.247
**Style length (cm)**	**1**	**6.09**	**0.014**
Stamen length (cm)	1	1.17	0.280
Herkogamy	1	0.68	0.408
Replicate	1	0.23	0.634

Supplementary Table 3: Impacts of pollination (bee-pollination or hand-pollination), herbivory (aphid-herbivory or no herbivory), and soil (limestone or tuff soil), and their interactions on plant-trait evolution, as estimated by two-sided linear mixed models (LMM) for plants of the two soil lines grown in their local soils (for reciprocal transplant data see Supplementary Table 5). Bold indicate significant factors effects (P<0.05) in driving divergence between treatments. (+) indicates positive effect (increase) of either bees or herbivory on plant traits evolution whereas (-) indicate a negative effect (decrease).

Traits	Factor	df	Chisq	P	Sign
Number of open flowers	**Soil lines (Sl)**	**1,611**	**41.72**	**$1.053^{*}10^{-10}$**	+
	Pollination (P)	1,611	1.35	0.250	
	**Herbivory (H)**	**1,611**	**10.73**	**0.010**	+
	Replicate	1,611	1.09	0.300	
	(Sl) x (H)	1,611	0.59	0.450	
	**(Sl) x (P)**	**1,611**	**5.66**	**0.020**	
	(P) x (H)	1,611	0.38	0.540	
	(Sl) x (P) x (H)	1,611	1.46	0.230	
Height at day 30 (cm)	**Soil lines (Sl)**	**1,612**	**156.22**	**$7.598^{*}10^{-36}$**	+
	Pollination (P)	1,612	0.99	0.330	
	Herbivory (H)	1,612	2.43	0.120	
	Replicate	1,612	2.07	0.150	
	(Sl) x (H)	1,612	1.22	0.270	
	**(Sl) x (P)**	**1,612**	**11.97**	**0.001**	
	**(P) x (H)**	**1,612**	**9.89**	**0.002**	
	**(Sl) x (P) x (H)**	**1,612**	**7.35**	**0.006**	

Leaf size (cm^2)	**Soil lines (Sl)**	**1,612**	**138.34**	**$6.153^{*}10^{-32}$**	+
	**Pollination (P)**	**1,612**	**6.70**	**0.010**	+
	**Herbivory (H)**	**1,612**	**16.74**	**$4.286^{*}10^{-05}$**	-
	Replicate	1,612	0.37	0.550	
	(Sl) x (H)	1,612	0.82	0.370	
	**(Sl) x (P)**	**1,612**	**16.06**	**$6.142^{*}10^{-05}$**	
	(P) x (H)	1,612	0.00	0.980	
	(Sl) x (P) x (H)	1,612	0.10	0.760	
Length per branch (cm)	**Soil lines (Sl)**	**1,612**	**75.25**	**$4.154^{*}10^{-18}$**	-
	Pollination (P)	1,612	0.11	0.738	
	Herbivory (H)	1,612	2.57	0.109	
	Replicate	1,612	0.54	0.463	
	(Sl) x (H)	1,612	0.30	0.586	
	(Sl) x (P)	1,612	2.63	0.105	
	(P) x (H)	1,612	1.12	0.289	
	**(Sl) x (P) x (H)**	**1,612**	**3.97**	**0.046**	
Time to first flower (day)	Soil lines (Sl)	1,618	1.09	0.300	
	Pollination (P)	1,618	0.03	0.870	
	**Herbivory (H)**	**1,618**	**32.14**	**$1.431^{*}10^{-08}$**	-
	Replicate	1,618	0.00	0.980	
	(Sl) x (H)	1,618	0.99	0.320	
	**(Sl) x (P)**	**1,618**	**25.05**	**$5.582^{*}10^{-07}$**	
	**(P) x (H)**	**1,618**	**6.06**	**0.020**	
	(Sl) x (P) x (H)	1,618	0.00	0.990	
Flower production	**Soil lines (Sl)**	**1,611**	**204.20**	**$2.535^{*}10^{-46}$**	+
	Pollination (P)	1,611	0.16	0.690	
	**Herbivory (H)**	**1,611**	**6.21**	**0.020**	+
	Replicate	1,611	0.35	0.560	
	(Sl) x (H)	1,611	2.26	0.140	
	**(Sl) x (P)**	**1,611**	**4.58**	**0.040**	
	(P) x (H)	1,611	2.72	0.100	
	(Sl) x (P) x (H)	1,611	0.42	0.520	
Flower diameter (cm)	**Soil lines (Sl)**	**1,515**	**15.18**	**$9.784^{*}10^{-5}$**	+
	**Pollination (P)**	**1,515**	**12.54**	**$3.981^{*}10^{-4}$**	+
	Herbivory (H)	1,515	0.37	0.550	
	Replicate	1,515	1.32	0.260	
	(Sl) x (H)	1,515	0.03	0.870	
	(Sl) x (P)	1,515	2.97	0.090	
	(P) x (H)	1,515	0.04	0.850	
	**(Sl) x (P) x (H)**	**1,515**	**6.33**	**0.020**	
Petal length (cm)	**Soil lines (Sl)**	**1,518**	**10.80**	**0.010**	+
	Pollination (P)	1,518	0.77	0.390	

	**Herbivory (H)**	**1,518**	**8.74**	**0.010**	+
	Replicate	1,518	0.89	0.350	
	(Sl) x (H)	1,518	0.27	0.610	
	(Sl) x (P)	1,518	3.77	0.060	
	(P) x (H)	1,518	1.34	0.250	
	(Sl) x (P) x (H)	1,518	0.12	0.730	
Petal width (cm)	**Soil lines (Sl)**	**1,518**	**5.62**	**0.020**	+
	**Pollination (P)**	**1,518**	**22.78**	**$1.813^{*}10^{-06}$**	+
	Herbivory (H)	1,518	1.01	0.320	
	Replicate	1,518	0.22	0.640	
	(Sl) x (H)	1,518	2.91	0.090	
	(Sl) x (P)	1,518	0.86	0.360	
	(P) x (H)	1,518	1.70	0.200	
	**(Sl) x (P) x (H)**	**1,518**	**5.14**	**0.030**	
Style length (cm)	**Soil lines (Sl)**	**1,518**	**6.39**	**0.020**	+
	Pollination (P)	1,518	0.80	0.380	
	**Herbivory (H)**	**1,518**	**4.20**	**0.050**	-
	Replicate	1,518	0.49	0.490	
	(Sl) x (H)	1,518	1.02	0.320	
	**(Sl) x (P)**	**1,518**	**9.28**	**0.010**	
	(P) x (H)	1,518	2.45	0.120	
	(Sl) x (P) x (H)	1,518	0.25	0.620	
Stamen length (cm)	**Soil lines (Sl)**	**1,518**	**19.89**	**$8.199^{*}10^{-06}$**	+
	Pollination (P)	1,518	0.31	0.590	
	**Herbivory (H)**	**1,518**	**15.04**	**$1.052^{*}10^{-04}$**	+
	Replicate	1,518	1.70	0.200	
	(Sl) x (H)	1,518	0.69	0.410	
	(Sl) x (P)	1,518	0.01	0.920	
	**(P) x (H)**	**1,518**	**5.24**	**0.030**	
	(Sl) x (P) x (H)	1,518	0.52	0.470	
Herkogamy	Soil lines (Sl)	1,518	1.49	0.230	
	Pollination (P)	1,518	0.04	0.850	
	Herbivory (H)	1,518	0.81	0.370	
	Replicate	1,518	2.06	0.160	
	(Sl) x (H)	1,518	3.34	0.070	
	**(Sl) x (P)**	**1,518**	**9.10**	**0.010**	
	(P) x (H)	1,518	2.86	0.100	
	(Sl) x (P) x (H)	1,518	2.92	0.090	
Bumblebee first choices (No-herbivory plants)	Soil lines (Sl)	1, 232	2.82	0.093	
	**Pollination (P)**	**1, 232**	**4.85**	**0.028**	+
	Replicate	1, 232	0.00	0.966	
	(Sl) x (P)	1, 232	0.08	0.779	

Bumblebee first choices (Herbivory plants)	**Soil lines (Sl)**	**1, 223**	**4.25**	**0.039**	**+**
	**Pollination (P)**	**1, 223**	**8.48**	**0.004**	**+**
	Replicate	1, 223	2.87	0.090	
	(Sl) x (P)	1, 223	0.00	0.980	

Supplementary Table 4: Impacts of herbivory (aphid herbivory or no herbivory), pollination (bee-pollination or hand-pollination) on the evolution of traits in plants of generation ten in each soil line separately (limestone and tuff), estimated by two-sided linear mixed models (LMM). Bold indicate significant factor effects (P<0.05). (+) indicates positive effect (increase) of either bees or herbivory on plant traits evolution whereas (-) indicate a negative effect (decrease).

			Limestone lines				Tuff lines			
Traits	Factor	df	N	Chsq	P	sign	N	Chsq	P	sign
MORPHOLOGY										
**Number of open flowers**	Pollination	1	299	1.00	0.317		313	**4.93**	**0.026**	+
	Herbivory	1		**11.14**	**$8.447^{*}10^{-04}$**	+		2.50	0.114	
	Replicate	1		1.01	0.315			2.62	0.105	
	Pollination x Herbivory	1		0.28	0.597			1.31	0.253	
**Height at day 30 (cm)**	Pollination	1	299	**14.13**	**0.002**	-	313	2.45	0.118	
	Herbivory	1		0.09	0.758			2.86	0.091	
	Replicate	1		0.00	0.993			3.29	0.070	
	Pollination x Herbivory	1		0.07	0.790			**14.31**	**$1.547^{*}10^{-04}$**	
**Leaf size (cm^2)**	Pollination	1	299	2.64	0.104		313	**14.27**	**$1.588*10^{-04}$**	+
	Herbivory	1		**10.74**	**0.001**	-		**8.25**	**0.004**	-
	Replicate	1		0.12	0.729			0.20	0.657	
	Pollination x Herbivory	1		0.11	0.733			0.03	0.872	
**Length per branch (cm)**	Pollination	1	299	**5.59**	**0.018**	+	313	0.51	0.475	
	Herbivory	1		**6.09**	**0.014**	+		0.35	0.556	
	Replicate	1		1.25	0.263			0.04	0.848	
	Pollination x Herbivory	1		1.22	0.269			2.91	0.088	
**Time to first flower (day)**	Pollination	1	302	**10.62**	**$8.253^{*}10^{-04}$**	-	316	**15.11**	**$1.492^{*}10^{-04}$**	+
	Herbivory	1		**9.14**	**0.003**	-		**24.60**	**$7.281^{*}10^{-07}$**	-
	Replicate	1		0.04	0.833			0.10	0.757	
	Pollination x Herbivory	1		2.26	0.133			3.24	0.072	
**Flower production**	Pollination	1	299	2.63	0.105		313	2.34	0.126	
	Herbivory	1		**12.91**	**$3.273^{*}10^{-04}$**	+		0.41	0.522	
	Replicate	1		0.46	0.497			0.04	0.850	
	Pollination x Herbivory	1		**4.15**	**0.042**			0.39	0.532	
**Flower diameter (cm)**	Pollination	1	245	1.46	0.226		270	**13.77**	**$2.061^{*}10^{-04}$**	+
	Herbivory	1		0.09	0.765			0.29	0.590	
	Replicate	1		0.73	0.394			0.59	0.444	
	Pollination x Herbivory	1		2.90	0.088			3.45	0.063	
**Petal length (cm)**	Pollination	1	247	0.68	0.408		271	3.60	0.058	
	Herbivory	1		2.81	0.094			**5.74**	**0.017**	+
	Replicate	1		0.01	0.905			0.46	0.496	
	Pollination x Herbivory	1		0.36	0.550			1.09	0.296	
**Petal width (cm)**	Pollination	1	247	**15.90**	**$6.676^{*}10^{-05}$**	+	271	**7.75**	**0.005**	+
	Herbivory	1		3.80	0.051			0.23	0.631	
	Replicate	1		0.02	0.902			0.02	0.895	
	Pollination x Herbivory	1		**6.43**	**0.011**			0.39	0.531	
**Style length (cm)**	Pollination	1	247	**9.70**	**0.002**	+	271	1.92	0.166	
	Herbivory	1		0.61	0.433			**4.00**	**0.045**	-
	Replicate	1		0.05	0.825			0.04	0.850	
	Pollination x Herbivory	1		0.62	0.431			1.89	0.169	
**Stamen length (cm)**	Pollination	1	247	0.07	0.786		271	0.22	0.643	
	Herbivory	1		**10.22**	**0.001**	+		**5.00**	**0.025**	+
	Replicate	1		1.96	0.162			0.01	0.923	
	Pollination x Herbivory	1		1.23	0.268			**4.66**	**0.031**	
Herkogamy	Pollination	1	247	**6.36**	**0.012**	+	271	3.44	0.064	

Herbivory	1	0.48	0.489	3.15	0.076
Replicate	1	0.67	0.414	0.22	0.637
Pollination x Herbivory	1	0.00	0.948	**5.15**	**0.023**

Supplementary Table 5: **Effects of soil, soil lines, biotic treatments (i.e. the treatments NHH, HH, NHB, HB, see Figure 2), the interaction of soil and soil lines, as well as the interaction of soil x soil lines x biotic treatments, in the reciprocal transplant experiment assessed by a general linear model.** Significances were determined using two-sided generalized mixed models using soil, soil lines, biotic treatments, and their interactions were included as fixed factors, and replicate as random factor. Bold indicates statistical significance. G x E interaction is indicated by a significant interaction of the factors soil (i.e. the soil the plants were cultivated in at the time of phenotyping) and soil lines (i.e. the soil the plants evolved in). The biotic treatments pollination and herbivory were combined to represent the analysis shown in Figure 2, where the combined effects of pollination and herbivory had the strongest effects on local adaptation. Traits shown in bold are those were the factors (except replicate) and all interactions are significant, thus, patterns of local adaption differ among biotic treatment groups.

Traits	Factor	Df	F	P
**Number of open flowers**	**Soil**	**1, 1212**	**39.43**	**$3.404^{*}10^{-10}$**
	**Soil lines**	**1, 1212**	**10.23**	**0.001**
	**Biotic treatment**	**3, 1212**	**8.37**	**0.039**
	Replicate	1, 1212	1.41	0.236
	**Soil x Soil lines**	**1, 1212**	**10.26**	**0.001**
	**Biotic treatment x Soil x Soil**	**9, 1212**	**22.03**	**0.009**
**Height at day 30 (cm)**	**Soil**	**1, 1214**	**128.54**	**$8.555^{*}10^{-30}$**
	**Soil lines**	**1, 1214**	**42.19**	**$8.301^{*}10^{-11}$**
	**Biotic treatment**	**3, 1214**	**31.21**	**$7.669^{*}10^{-07}$**
	Replicate	1, 1214	1.51	0.219
	**Soil x Soil lines**	**1, 1214**	**8.93**	**0.003**
	**Biotic treatment x Soil x Soil**	**9, 1214**	**42.76**	**$2.388^{*}10^{-06}$**
**Leaf size (cm^2)**	**Soil**	**1, 1213**	**84.03**	**$4.879^{*}10^{-20}$**
	**Soil lines**	**1, 1213**	**71.22**	**$3.200^{*}10^{-17}$**
	**Biotic treatment**	**3, 1213**	**47.47**	**$2.765^{*}10^{-10}$**
	Replicate	1, 1213	0.23	0.630
	**Soil x Soil lines**	**1, 1213**	**5.99**	**0.014**
	**Biotic treatment x Soil x Soil**	**9, 1213**	**49.52**	**$1.325^{*}10^{-07}$**
Length per branch (cm)	**Soil**	**1, 1213**	**155.29**	**$1.210^{*}10^{-35}$**
	Soil lines	1, 1213	0.63	0.426
	**Biotic treatment**	**3, 1213**	**10.37**	**0.016**
	Replicate	1, 1213	0.11	0.742
	Soil x Soil lines	1, 1213	0.04	0.848
	Biotic treatment x Soil x Soil lines	9, 1213	15.63	0.075
**Time to first flower (day)**	**Soil**	**1, 1236**	**104.81**	**$1.345^{*}10^{-24}$**
	**Soil lines**	**1, 1236**	**75.79**	**$3.152^{*}10^{-18}$**
	**Biotic treatment**	**3, 1236**	**47.91**	**$2.221^{*}10^{-10}$**
	Replicate	1, 1236	0.07	0.789
	**Soil x Soil lines**	**1, 1236**	**3.61**	**0.050**

	**Biotic treatment x Soil x Soil**	**9, 1236**	**91.49**	**$8.187^{*}10^{-16}$**
Flower production	**Soil**	**1, 1212**	**440.08**	**$1.039^{*}10^{-97}$**
	Soil lines	1, 1212	1.06	0.303
	**Biotic treatment**	**3, 1212**	**19.34**	**$2.320^{*}10^{-04}$**
	Replicate	1, 1212	1.13	0.288
	**Soil x Soil lines**	**1, 1212**	**5.44**	**0.020**
	**Biotic treatment x Soil x Soil**	**9, 1212**	**51.37**	**$5.935^{*}10^{-08}$**
Flower diameter (cm)	**Soil**	**1, 1026**	**4.55**	**0.033**
	**Soil lines**	**1, 1026**	**9.50**	**0.002**
	**Biotic treatment**	**3, 1026**	**18.63**	**$3.267^{*}10^{-04}$**
	Replicate	1, 1026	1.39	0.239
	Soil x Soil lines	1, 1026	0.60	0.440
	**Biotic treatment x Soil x Soil**	**9, 1026**	**22.68**	**0.007**
Petal length (cm)	Soil	1, 1031	1.41	0.235
	**Soil lines**	**1, 1031**	**11.66**	**$6.370^{*}10^{-04}$**
	Biotic treatment	3, 1031	4.33	0.228
	Replicate	1, 1031	0.91	0.341
	Soil x Soil lines	1, 1031	0.17	0.681
	**Biotic treatment x Soil x Soil**	**9, 1031**	**21.49**	**0.011**
Petal width (cm)	**Soil**	**1, 1031**	**6.83**	**0.009**
	Soil lines	1, 1031	0.31	0.575
	**Biotic treatment**	**3, 1031**	**46.47**	**$4.507^{*}10^{-10}$**
	Replicate	1, 1031	0.96	0.327
	Soil x Soil lines	1, 1031	0.64	0.425
	**Biotic treatment x Soil x Soil**	**9, 1031**	**20.28**	**0.016**
Style length (cm)	**Soil**	**1, 1031**	**10.09**	**0.001**
	Soil lines	1, 1031	0.11	0.737
	Biotic treatment	3, 1031	1.94	0.585
	Replicate	1, 1031	0.73	0.394
	Soil x Soil lines	1, 1031	0.21	0.648
	**Biotic treatment x Soil x Soil**	**9, 1031**	**25.71**	**0.002**
Stamen length (cm)	Soil	1, 1031	2.90	0.088
	**Soil lines**	**1, 1031**	**18.30**	**$1.884^{*}10^{-05}$**
	**Biotic treatment**	**3, 1031**	**19.38**	**$2.283^{*}10^{-04}$**
	Replicate	1, 1031	1.75	0.185
	Soil x Soil lines	1, 1031	0.01	0.906
	Biotic treatment x Soil x Soil lines	9, 1031	10.55	0.308
Herkogamy	Soil	1, 1031	2.94	0.087
	Soil lines	1, 1031	0.00	0.978
	Biotic treatment	3, 1031	2.07	0.557
	Replicate	1, 1031	1.74	0.187

Soil x Soil lines	1, 1031	0.58	0.446
**Biotic treatment x Soil x Soil**	**9, 1031**	**24.01**	**0.004**

Supplementary Table 6: Trait differences (mean ± SD) among plants of generation one and ten, having evolved with herbivory and bee pollination when growing on both tuff and limestone. Significant differences with generation one (for plants growing on same soil type) are indicated below each treatment group mean. Significance between treatments was determined using two-sided linear mixed models (LMM) with individual traits as dependent variable, soil, soil lines and their interaction as fixed factor, and replicate as random factors. Bold indicate significant factor effects (P<0.05) and traits that showed a significant G x E interaction where G is represented by the factor "Soil lines" (i.e. the soil the plant evolved in; the plant genotype) and E by "Soil" (the soil the plants were cultivated on at the time of phenotyping; environment); this analysis did not include plants of generation one. When Soil and Soil lines interaction was significant multiple-comparison post hoc tests were run to compare local versus foreign and home versus away contrasts using estimated marginal means (EMMs) and their linear contrasts (emmeans package: Lenth 2021; Statistical differences (P<0.05) are indicated in bold). **LG1**, **TG1**: plants of generation one growing in limestone (L) or tuff (T) soil. **LLHB**: limestone line plants growing in limestone (LL), with herbivory (H) and bee pollination (B). **TLHB**: tuff line plants growing in limestone (TL), with herbivory (H) and bee pollination (B). **LTHB**: limestone line plants growing in tuff (LT) with herbivory (H) and bee pollination (B). **TTHB**: tuff line plants growing in tuff (TT) with herbivory (H) and bee pollination (B).

Traits	LG1	TG1	N	LLHB	TLHB	LTHB	TTHB	Factor	df	Chsq	P
MORPHOLOGY											
**Number of open flowers**	**5.63 ± 3.81**	**6.58 ± 4.28**	**298**	**6.89 ± 4.83**	**6.43 ± 3.88**	**7.09 ± 5.88**	**11.04 ± 7.22**	**Soil (S)**	**1**	**14.17**	**1.667*10^{-04}**
							6.246*10^{-06}	**Soil lines (Sl)**	**1**	**7.06**	**0.008**
								Replicate	1	0.06	0.803
								**S x Sl**	**1**	**11.50**	**6.960*10^{-04}**
Height at day 30 (cm)	33.10 ± 6.57	35.19 ± 5.94	299	29.44 ± 7.24	29.14 ± 6.54	33.26 ± 9.21	36.34 ± 7.94	**Soil (S)**	**1**	**37.90**	**7.455*10^{-10}**
				0.001	2.574*10^{-04}			Soil lines (Sl)	1	2.35	0.125
								Replicate	1	0.09	0.766
								S x Sl	1	3.54	0.060
Leaf size (cm^2)	8.18 ± 3.31	7.73 ± 2.55	298	6.12±2.17	8.49±3.32	7.42±3.45	10.71±4.46	**Soil (S)**	**1**	**19.73**	**8.931*10^{-06}**
				5.608*10^{-06}			7.798*10^{-07}	**Soil lines (Sl)**	**1**	**50.19**	**1.393*10^{-12}**
								Replicate	1	0.44	0.507
								S x Sl	1	1.27	0.260
Length per branch (cm)	3.68 ± 1.52	6.46 ± 6.81	298	4.65 ± 3.65	1.96 ± 3.75	8.52 ± 6.38	6.67 ± 6.81	**Soil (S)**	**1**	**48.14**	**3.961*10^{-12}**
				0.004		0.002		**Soil lines (Sl)**	**1**	**13.47**	**2.423*10^{-04}**
								Replicate	1	0.01	0.917
								S x Sl	1	0.47	0.492
**Time to first flower (day)**	**22.08 ± 1.74**	**21.46 ± 1.45**	**306**	**21.45 ± 2.32**	**24.64 ± 2.69**	**20.20 ± 1.90**	**22.00 ± 2.77**	**Soil (S)**	**1**	**49.76**	**1.734*10^{-12}**
					3.979*10^{-12}	1.183*10^{-06}		**Soil lines (Sl)**	**1**	**79.90**	**3.932*10^{-19}**
								Replicate	1	1.45	0.229
								**S x Sl**	**1**	**7.21**	**0.007**
Flower production	21.61 ± 6.59	28.26 ± 6.50	298	22.63 ± 9.12	18.89 ± 6.59	34.55 ± 13.67	34.64 ± 12.20	**Soil (S)**	**1**	**124.50**	**6.544*10^{-29}**
					0.011	4.296*10^{-04}	5.029*10^{-05}	Soil lines (Sl)	1	2.18	0.140
								Replicate	1	0.05	0.828
								S x Sl	1	2.34	0.127
Flower diameter (cm)	1.35 ± 0.17	1.31 ± 0.13	261	1.32 ± 0.17	1.35 ± 0.17	1.31 ± 0.20	1.37 ± 0.17	Soil (S)	1	0.00	0.975
							0.025	**Soil lines (Sl)**	**1**	**4.27**	**0.039**
								Replicate	1	0.17	0.677
								S x Sl	1	0.40	0.527
**Petal length (cm)**	**0.94 ± 0.15**	**0.95 ± 0.12**	**261**	**0.90 ± 0.15**	**0.94 ± 0.13**	**0.87 ± 0.17**	**0.98 ± 0.21**	Soil (S)	1	0.15	0.694

						$4.649^{*}10^{-04}$		**Soil lines (Sl)**	**1**	**14.26**	**$1.589*10^{-04}$**
								Replicate	1	0.28	0.600
								**S x Sl**	**1**	**2.87**	**0.004**
Petal width (cm)	0.45 ± 0.06	0.43 ± 0.06	261	0.45 ± 0.06	0.45 ± 0.06	0.45 ± 0.08	0.46 ± 0.06	Soil (S)	1	0.40	0.526
							0.006	Soil lines (Sl)	1	0.00	0.954
								Replicate	1	0.02	0.876
								S x Sl	1	0.51	0.477
Style length (cm)	0.75 ± 0.17	0.80 ± 0.16	261	0.72 ± 0.17	0.76 ± 0.19	0.78 ± 0.19	0.82 ± 0.21	**Soil (S)**	**1**	**6.60**	**0.010**
								Soil lines (Sl)	1	3.18	0.074
								Replicate	1	0.54	0.462
								S x Sl	1	0.08	0.771
Stamen length (cm)	0.63 ± 0.06	0.63 ± 0.06	261	0.61 ± 0.06	0.63 ± 0.08	0.60 ± 0.10	0.64 ± 0.07	Soil (S)	1	0.01	0.905
								**Soil lines (Sl)**	**1**	**8.37**	**0.004**
								Replicate	1	1.21	0.271
								S x Sl	1	0.56	0.452
Herkogamy	0.18 ± 0.11	0.20 ± 0.13	261	0.18 ± 0.10	0.19 ± 0.13	0.22 ± 0.14	0.23 ± 0.12	**Soil (S)**	**1**	**7.85**	**0.005**
								Soil lines (Sl)	1	0.31	0.576
								Replicate	1	0.36	0.546
								S x Sl	1	0.02	0.899

Post hoc tests

	Parameters	Treatments	n	t – value	P
**Nb flowers**	**« foreign » versus « local »**	**HB**	**298**	**-3.39**	**$7.916^{*}10^{-04}$**
	**« home » versus « away »**	**HB**	**298**	**2.68**	**0.008**
**Time to first flower**	**"foreign" versus "local"**	**HB**	**306**	**2.69**	**0.008**
	**« home » versus "away"**	**HB**	**306**	**8.94**	**$4.054^{*}10^{-17}$**
Petal length	"foreign" versus "local"	HB	261	-1.69	0.092
	**"home" versus "away"**	**HB**	**261**	**3.81**	**$1.770^{*}10^{-04}$**
Plant height at day 30	"foreign" versus "local"	HB	299	-1.88	0.061
	"home" versus "away"	HB	299	1.54	0.125

Supplementary Table 7: Trait differences (mean ± SD) among plants of generation one and ten, having evolved with herbivory and hand-pollination when growing on both tuff and limestone. Significant differences with generation one (for plants growing on same soil type) are indicated below each treatment group mean. Significance between treatments was determined using two-sided linear mixed models (LMM) with individual traits as dependent variable, soil, soil lines and their interaction as fixed factor, and replicate as random factors. Bold indicate significant factor effects (P<0.05) and traits that showed a significant G x E interaction where G is represented by the factor "Soil lines" (i.e. the soil the plant evolved in; the plant genotype) and E by "Soil" (the soil the plants were cultivated on at the time of phenotyping; the environment); this analysis did not include plants of generation one. When Soil and Soil lines interaction was significant multiple-comparison post hoc tests were run to compare local versus foreign and home versus away contrasts using estimated marginal means (EMMs) and their linear contrasts (emmeans package; Statistical differences (P<0.05) are indicated in bold). **LG1**, **TG1**: plants of generation one growing in limestone (L) or tuff (T) soil. **LLHH**: limestone line plants growing in limestone (LL), with herbivory (H) and hand-pollination (H). **TLHH**: tuff line plants growing in limestone (TL), with herbivory (H) and hand-pollination (H). **LTHH**: limestone line plants growing in tuff (LT) with herbivory (H) and hand-pollination (H); **TTHH**: tuff line plants growing in tuff (TT) with herbivory (H) and hand-pollination (H).

Traits	LG1	TG1	N	LLHH	TLHH	LTHH	TTHH	Factor	df	Chsq	P
MORPHOLOGY											
Number of open flowers	5.63 ± 3.81	6.58 ± 4.28	296	7.69 ± 5.58	5.72 ± 3.66	8.69 ± 6.66	8.66 ± 5.88	**Soil (S)**	**1**	**9.79**	**0.002**
				0.009		0.025	0.015	Soil lines (Sl)	1	2.60	0.107
								Replicate	1	1.09	0.297
								S x Sl	1	2.34	0.127
**Height at day 30 (cm)**	**33.10 ± 6.57**	**35.19 ± 5.94**	**297**	**32.27 ± 5.76**	**31.67 ± 7.94**	**35.46 ± 7.27**	**38.33 ± 9.08**	**Soil (S)**	**1**	**36.03**	**$1.947*10^{-09}$**
							0.006	Soil lines (Sl)	1	2.01	0.156
								Replicate	1	4.86	0.275
								**S x Sl**	**1**	**4.25**	**0.039**
Leaf size (cm^2)	8.18 ± 3.31	7.73 ± 2.55	297	6.68 ± 2.58	6.61 ± 2.92	8.35 ± 3.08	8.66 ± 2.92	**Soil (S)**	**1**	**26.84**	**$2.211*10^{-07}$**
				0.003	0.002			Soil lines (Sl)	1	0.14	0.711
								Replicate	1	3.78	0.052
								S x Sl	1	0.25	0.617
Length per branch (cm)	3.68 ± 1.52	6.46 ± 6.81	296	3.32 ± 3.46	4.60 ± 4.95	8.03 ± 8.78	8.61 ± 7.05	**Soil (S)**	**1**	**34.44**	**$4.404*10^{-09}$**
							0.025	Soil lines (Sl)	1	1.56	0.212
								Replicate	1	0.02	0.881
								S x Sl	1	0.22	0.636
Time to first flower (day)	22.08 ± 1.74	21.46 ± 1.45	303	21.90 ± 2.38	22.10 ± 2.38	21.10 ± 1.80	20.58 ± 2.22	**Soil (S)**	**1**	**24.14**	**$8.954*10^{-07}$**
							0.004	Soil lines (Sl)	1	0.29	0.588
								**Replicate**	**1**	**4.41**	**0.036**
								S x Sl	1	2.23	0.136
Flower production	21.61 ± 6.59	28.26 ± 6.50	296	25.81 ± 8.20	19.78 ± 7.77	38.72 ± 12.50	33.51 ± 10.97	**Soil (S)**	**1**	**130.49**	**$3.194*10^{-30}$**
				$6.593*10^{-04}$		$3.620*10^{-10}$	$2.709*10^{-04}$	**Soil lines (Sl)**	**1**	**23.27**	**$1.408*10^{-06}$**
								Replicate	1	0.07	0.786
								S x Sl	1	0.12	0.726
Flower diameter (cm)	1.34 ± 0.17	1.31 ± 0.13	242	1.27 ± 0.14	1.31 ± 0.17	1.30 ± 0.14	1.33 ± 0.18	Soil (S)	1	1.48	0.224
				0.005				Soil lines (Sl)	1	3.28	0.100
								Replicate	1	0.60	0.439

								S x Sl	1	0.02	0.875
Petal length (cm)	0.94 ± 0.15	0.95 ± 0.12	242	0.90 ± 0.12	0.91 ± 0.14	0.88 ± 0.15	0.92 ± 0.16	Soil (S)	1	0.12	0.724
						0.004		Soil lines (Sl)	1	1.43	0.233
								Replicate	1	1.55	0.213
								S x Sl	1	1.11	0.292
Petal width (cm)	0.45 ± 0.06	0.43 ± 0.06	242	0.40 ± 0.05	0.43 ± 0.05	0.41 ± 0.06	0.44 ± 0.06	Soil (S)	1	2.24	0.135
				$8.416^{*}10^{-06}$	0.049	0.047		**Soil lines (Sl)**	**1**	**16.78**	**$4.209*10^{-05}$**
								Replicate	1	0.19	0.661
								S x Sl	1	0.06	0.805
**Style length (cm)**	**0.75 ± 0.17**	**0.80 ± 0.16**	**242**	**0.80 ± 0.17**	**0.71 ± 0.17**	**0.79 ± 0.17**	**0.82 ± 0.20**	**Soil (S)**	**1**	**5.05**	**0.025**
								Soil lines (Sl)	1	2.23	0.135
								Replicate	1	0.01	0.936
								**S x Sl**	**1**	**6.53**	**0.011**
Stamen length (cm)	0.63 ± 0.06	0.63 ± 0.06	242	0.60 ± 0.06	0.59 ± 0.07	0.61 ± 0.07	0.62 ± 0.07	Soil (S)	1	3.45	0.063
				0.007	$7.495^{*}10^{-04}$			Soil lines (Sl)	1	0.07	0.795
								Replicate	1	0.01	0.732
								S x Sl	1	1.71	0.191
Herkogamy	0.18 ± 0.11	0.20 ± 0.13	242	0.21 ± 0.14	0.18 ± 0.12	0.20 ± 0.13	0.24 ± 0.14	Soil (S)	1	1.08	0.298
								Soil lines (Sl)	1	0.02	0.893
								Replicate	1	0.37	0.541
								**S x Sl**	**1**	**3.88**	**0.049**

Post hoc tests

	Parameters	Treatments	n	t – value	P
**Plant height at day 30 (cm)**	**"foreign" versus "local"**	**HH**	**297**	**-2.06**	**0.042**
	"home" versus "away"	HH	297	1.41	0.159
**Style length (cm)**	**"foreign" versus "local"**	**HH**	**242**	**-2.56**	**0.011**
	"home" versus "away"	HH	242	-1.41	0.160

Supplementary Table 8: Trait differences (mean ± SD) among plants of generation one and ten, having evolved without herbivory, with bee pollination when growing on both tuff and limestone. Significant differences with generation one (for plants growing on same soil type) are indicated below each treatment group mean. Significance between treatments was determined using two-sided linear mixed models (LMM) with individual traits as dependent variable, soil, soil lines and their interaction as fixed factor, and replicate as random factors. Bold indicate significant factor effects ($P<0.05$) and traits that showed a significant G x E interaction where G is represented by the factor "Soil lines" (i.e. the soil the plant evolved in; the plant genotype) and E by "Soil" (the soil the plants were cultivated on at the time of phenotyping; the environment); this analysis did not include plants of generation one. When Soil and Soil lines interaction was significant multiple-comparison post hoc tests were run to compare local versus foreign and home versus away contrasts using estimated marginal means (EMMs) and their linear contrasts (emmeans package: Lenth 2021; Statistical differences ($P<0.05$) are indicated in bold). **LG1**, **TG1**: plants of generation one growing in limestone (L) or tuff (T) soil. **LLNHB**: limestone line plants growing in limestone (LL), without herbivory (NH) and bee pollination (B). **TLNHB**: tuff line plants growing in limestone (TL), without herbivory (NH) and bee pollination (B). **LTNHB**: limestone line plants growing in tuff (LT) without herbivory (NH) and bee pollination (B). **TTNHB**: tuff line plants growing in tuff (TT) without herbivory (NH) and bee pollination (B).

Traits	LG1	TG1	N	LLNHB	TLNHB	LTNHB	TTNHB	Factor	df	Chsq	P
MORPHOLOGY											
Number of open flowers	5.63 ± 3.81	6.58 ± 4.28	305	5.41 ± 4.28	6.85 ± 4.22	6.49 ± 6.06	9.06 ± 6.16	**Soil (S)**	**1**	**7.87**	**0.005**
							0.004	**Soil lines (Sl)**	**1**	**11.90**	**$5.628*10^{-04}$**
								Replicate	1	0.78	0.376
								S x Sl	1	0.92	0.339
Height at day 30 (cm)	33.10 ± 6.57	35.19 ± 5.94	305	29.84 ± 5.87	35.37 ± 6.43	33.12 ± 6.34	41.18 ± 7.70	**Soil (S)**	**1**	**37.52**	**$9.057*10^{-10}$**
				0.002	0.027	0.042	$7.493^{*}10^{-08}$	**Soil lines (Sl)**	**1**	**79.76**	**$4.230*10^{-19}$**
								Replicate	1	1.27	0.260
								S x Sl	1	2.81	0.954
Leaf size (cm^2)	8.18 ± 3.31	7.73 ± 2.55	305	7.17 ± 2.52	10.12 ± 3.71	8.38 ± 2.69	12.14 ± 4.79	**Soil (S)**	**1**	**16.18**	**$5.755*10^{-05}$**
				0.043	$7.155^{*}10^{-04}$		$3.504^{*}10^{-12}$	**Soil lines (Sl)**	**1**	**66.36**	**$3.750*10^{-16}$**
								Replicate	1	1.19	0.276
								S x Sl	1	0.98	0.323
Length per branch (cm)	3.68 ± 1.52	6.46 ± 6.81	305	3.23 ± 2.32	3.13 ± 4.19	7.93 ± 8.60	7.55 ± 7.41	**Soil (S)**	**1**	**42.09**	**$8.727*10^{-11}$**
								Soil lines (Sl)	1	0.09	0.763
								Replicate	1	0.17	0.678
								S x Sl	1	0.04	0.848

Time to first flower (day)	22.08 ± 1.74	21.46 ± 1.45	309	21.85 ± 1.69	24.05 ± 2.29	21.05 ± 1.38	22.79 ± 1.78	**Soil (S)**	**1**	**25.21**	**$5.149*10^{-07}$**
					$5.579^{*}10^{-08}$		$3.391^{*}10^{-07}$	**Soil lines (Sl)**	**1**	**87.14**	**$1.010*10^{-20}$**
								Replicate	1	0.54	0.445
								S x Sl	1	1.43	0.232
Flower production	21.61 ± 6.59	28.26 ± 6.50	305	21.31 ± 6.54	22.04 ± 7.14	30.00 ± 12.48	34.62 ± 10.63	**Soil (S)**	**1**	**97.31**	**$5.931*10^{-23}$**
							$1.472^{*}10^{-05}$	**Soil lines (Sl)**	**1**	**6.13**	**0.013**
								Replicate	1	0.06	0.812
								S x Sl	1	3.22	0.073
Flower diameter (cm)	1.35 ± 0.17	1.31 ± 0.13	252	1.28 ± 0.13	1.38 ± 0.14	1.33 ± 0.16	1.39 ± 0.13	Soil (S)	1	2.18	0.140
				0.021			$1.182^{*}10^{-05}$	**Soil lines (Sl)**	**1**	**21.61**	**$3.337*10^{-06}$**
								Replicate	1	1.13	0.287
								S x Sl	1	0.81	0.368
Petal length (cm)	0.94 ± 0.15	0.95 ± 0.12	254	0.86 ± 0.14	0.92 ± 0.15	0.91 ± 0.14	0.92 ± 0.14	Soil (S)	1	0.83	0.362
				0.002				**Soil lines (Sl)**	**1**	**4.08**	**0.043**
								Replicate	1	0.05	0.823
								S x Sl	1	2.20	0.138
Petal width (cm)	0.45 ± 0.06	0.43 ± 0.06	254	0.45 ± 0.06	0.45 ± 0.06	0.47 ± 0.08	0.46 ± 0.06	Soil (S)	1	3.15	0.076
						0.002	0.003	Soil lines (Sl)	1	0.19	0.666
								Replicate	1	0.89	0.345
								S x Sl	1	0.68	0.408
Style length (cm)	0.75 ± 0.17	0.80 ± 0.16	254	0.72 ± 0.17	0.79 ± 0.20	0.75 ± 0.20	0.80 ± 0.22	Soil (S)	1	1.07	0.300
								**Soil lines (Sl)**	**1**	**5.79**	**0.016**
								Replicate	1	2.52	0.113
								S x Sl	1	0.18	0.675
Stamen length (cm)	0.63 ± 0.06	0.63 ± 0.06	254	0.57 ± 0.10	0.61 ± 0.09	0.58 ± 0.09	0.60 ± 0.08	Soil (S)	1	0.07	0.792
				$1.112^{*}10^{-05}$		0.002	0.020	**Soil lines (Sl)**	**1**	**5.17**	**0.023**
								Replicate	1	0.35	0.554
								S x Sl	1	1.27	0.259
Herkogamy	0,18 ± 0,11	0,20 ± 0,13	254	0,19 ± 0,10	0,23 ± 0,15	0.20 ± 0.14	0.23 ± 0.16	Soil (S)	1	0.47	0.494
					0.035			**Soil lines (Sl)**	**1**	**4.32**	**0.038**
								Replicate	1	2.67	0.102

			S x Sl	1	0.00	0.975

Post hoc tests					
	Parameters	Treatments	n	t-value	P
Flower production	"foreign" versus "local"	NHB	305	-1.79	0.074
	**"home" versus "away"**	**NHB**	**305**	**2.48**	**0.014**

Supplementary Table 9: Traits differences (mean ± SD) among plants of generation one and ten, having evolved without herbivory, with hand-pollination when growing on both tuff and limestone. Significant differences with generation one (for plants growing on same soil type) are indicated below each treatment group mean. Significance between treatments was determined using two-sided linear mixed models (LMM) with individual traits as dependent variable, soil, soil lines and their interaction as fixed factor, and replicate as random factors. Bold indicate significant factor effects (P<0.05) and traits that showed a significant G x E interaction where G is represented by the factor "Soil lines" (i.e. the soil the plant evolved in; the plant genotype) and E by "soil" (the soil the plants were cultivated on at the time of phenotyping; the environment); this analysis did not include plants of generation one. When Soil and Soil lines interaction was significant multiple-comparison post hoc tests were run to compare local versus foreign and home versus away contrasts using estimated marginal means (EMMs) and their linear contrasts (emmeans package: Lenth 2021; Statistical differences (P<0.05) are indicated in bold). **LG1**, **TG1**: plants of generation one growing in limestone (L) or tuff (T) soil. **LLNHH**: limestone line plants growing in limestone (LL), without herbivory (NH) and hand-pollination (H). **TLNHH**: tuff line plants growing in limestone (TL), without herbivory (NH) and hand-pollination (H). **LTNHH**: limestone line plants growing in tuff (LT) without herbivory (NH) and hand-pollination (H). **TTNHH**: tuff line plants growing in tuff (TT) without herbivory (NH) and hand-pollination (H).

Traits	LG1	TG1	N	LLNHH	TLNHH	LTNHH	TTNHH	Factor	df	Chsq	P
MORPHOLOGY											
Number of open flowers	5.63 ± 3.81	6.58 ± 4.28	313	5.60 ± 4.16	6.45 ± 4.48	6.94 ± 5.32	8.30 ± 6.33	**Soil (S)**	**1**	**7.86**	**0.005**
								Soil lines (Sl)	1	3.67	0.055
								Replicate	1	1.60	0.206
								S x Sl	1	0.19	0.662
Height at day 30 (cm)	33.10 ± 6.57	35.19 ± 5.94	313	32.31 ± 5.25	33.32 ± 5.81	35.30 ± 6.29	36.56 ± 7.23	**Soil (S)**	**1**	**19.92**	**$8.089*10^{-06}$**
								Soil lines (Sl)	1	2.67	0.102
								Replicate	1	0.25	0.620
								S x Sl	1	0.03	0.856
**Leaf size (cm^2)**	**8.18 ± 3.31**	**7.73 ± 2.55**	**313**	**7.54 ± 2.74**	**7.20 ± 2.80**	**8.73 ± 4.10**	**10.25 ± 5.00**	**Soil (S)**	**1**	**26.85**	**$2.198*10^{-07}$**
					0.047		$1.239*10^{-4}$	Soil lines (Sl)	1	1.94	0.164
								Replicate	1	2.84	0.092
								**S x Sl**	**1**	**4.99**	**0.026**
Length per branch (cm)	3.68 ± 1.52	6.46 ± 6.81	313	2.78 ± 4.02	2.98 ± 3.54	6.00 ± 6.42	6.77 ± 6.75	**Soil (S)**	**1**	**33.91**	**$5.770*10^{-09}$**
								Soil lines (Sl)	1	0.661	0.416
								Replicate	1	0.634	0.426
								S x Sl	1	0.228	0.633
Time to first flower (day)	22.08 ± 1.74	21.46 ± 1.45	318	23.24 ± 3.08	23.08 ± 2.99	21.71 ± 1.92	22.26 ± 1.96	**Soil (S)**	**1**	**17.03**	**$3.673*10^{-05}$**
				0.002	0.010		0.004	Soil lines (Sl)	1	0.62	0.432
								Replicate	1	1.58	0.209
								S x Sl	1	1.47	0.226
Flower production	21.61 ± 6.59	28.26 ± 6.50	313	20.91 ± 6.30	22.19 ± 6.66	28.82 ± 9.22	31.92 ± 10.57	**Soil (S)**	**1**	**8.08**	**$6.285*10^{-21}$**
								**Soil lines (Sl)**	**1**	**5.37**	**0.021**
								Replicate	1	0.05	0.823
								S x Sl	1	0.94	0.333
Flower diameter (cm)	1.35 ± 0.17	1.31 ± 0.13	271	1.29 ± 0.16	1.28 ± 0.17	1.34 ± 0.18	1.29 ± 0.14	Soil (S)	1	2.94	0.087
					0.017			**Soil lines (Sl)**	**1**	**4.23**	**0.040**

								Replicate	1	4.38	0.363
								S x Sl	1	1.34	0.246
Petal length (cm)	0.94 ± 0.15	0.95 ± 0.12	274	0.88 ± 0.14	0.91 ± 0.13	0.94 ± 0.14	0.90 ± 0.15	Soil (S)	1	1.44	0.230
				0.022			0.033	Soil lines (Sl)	1	0.21	0.646
								Replicate	1	0.51	0.474
								**S x Sl**	**1**	**4.40**	**0.036**
Petal width (cm)	0.45 ± 0.06	0.43 ± 0.06	274	0.44 ± 0.06	0.43 ± 0.06	0.46 ± 0.06	0.43 ± 0.07	Soil (S)	1	2.26	0.133
						0.001		**Soil lines (Sl)**	**1**	**3.87**	**0.049**
								Replicate	1	0.19	0.664
								S x Sl	1	2.31	0.128
Style length (cm)	0.75 ± 0.17	0.80 ± 0.16	274	0.77 ± 0.18	0.73 ± 0.19	0.80 ± 0.17	0.74 ± 0.17	Soil (S)	1	0.54	0.463
							0.024	**Soil lines (Sl)**	**1**	**5.35**	**0.021**
								Replicate	1	0.00	0.978
								S x Sl	1	0.43	0.512
Stamen length (cm)	0.63 ± 0.06	0.63 ± 0.06	274	0.58 ± 0.08	0.61 ± 0.08	0.60 ± 0.09	0.62 ± 0.08	Soil (S)	1	2.35	0.126
				$2.662^{*}10^{-05}$		0.046		**Soil lines (Sl)**	**1**	**8.22**	**0.004**
								Replicate	1	2.95	0.086
								S x Sl	1	0.60	0.440
Herkogamy	0.18 ± 0.11	0.20 ± 0.13	274	0.23 ± 0.13	0.19 ± 0.11	0.22 ± 0.11	0.17 ± 0.11	Soil (S)	1	0.94	0.333
				0.018				**Soil lines (Sl)**	**1**	**10.58**	**0.001**
								Replicate	1	0.46	0.495
								S x Sl	1	0.09	0.764

Post hoc tests

	Parameters	Treatments	n	t-value	P
**Leaf size (cm^2)**	**"foreign" versus "local"**	NHH	313	**-2.32**	**0.026**
	"home" versus "away"	**NHH**	**313**	-1.41	0.159

Supplementary Table 10: Candidate genes associated with SNP markers with an evolutionary pattern of antagonistic pleiotropy. **HB:** plants that evolved with herbivory (H) and bee-pollination (B). **NHB:** plants that evolved without herbivory (NH) and bee-pollination (B).

Treatment	Gene	Chr	Position on chr	Transcript	Putative biological function (if known)
HB	LOC103866355	1	9223594	oligopeptide_transporter_5	
HB	LOC103837034	1	22918640	40S_ribosomal_protein_S20-2	Negative Regulators in ABA Signal Transduction; biotic and abiotic stress related genes such as stomata; pathogen defense [2]
HB	LOC117134232	1	22929496		
HB	LOC103837411	1	22952353	uncharacterized_LOC103837411	
HB	LOC103859104	3	15972395	TPR_repeat-containing_thioredoxin_TTL1	Involved in osmotic stress response [4]
HB	LOC103861268	3	25559370	NA	
HB	LOC103861878	3	28612153	zinc_finger_CCCH_domain-containing_protein_49	Involved in plant development and stress response [3]
HB	LOC103862024	3	29542224	U3_small_nucleolar_RNA-associated_protein_20%2C_transcript_variant_X1	
HB	LOC103862213	3	30310636	(S)-coclaurine_N-methyltransferase	
HB	LOC103870680	4	5297004	PH%2C_RCC1_and_FYVE_domains-containing_protein_1%2C_transcript_variant_X2	
HB	LOC117133775	4	5313554	uncharacterized_LOC117133775	
HB	LOC103870642	4	5326853	casein_kinase_1-like_protein_11%2C_transcript_variant_X2	Regulation flowering time [8]
HB	LOC103863673	4	5506432	agamous-like_MADS-box_protein_AGL97%2C_transcript_variant_X2	Defense, transcription factor [5]; regulation flowering time [7]
HB	LOC103863558	4	5522593	protein_transport_protein_SEC23	
HB	LOC103863574	4	5652383	heat_stress_transcription_factor_A-5%2C_transcript_variant_X2	
HB	LOC103832076	7	27118079	WD_repeat-containing_protein_DWA2	Negative Regulators in ABA Signal Transduction; biotic and abiotic stress related genes such as stomata; pathogen defense [2]
HB	LOC103832149	7	27445098	nuclear_speckle_RNA-binding_protein_A%2C_transcript_variant_X2	
HB	LOC103843072	9	42309072	disease_resistance_protein_RFL1	
NHB	LOC103851339	2	4144073	sucrose-phosphate_synthase_1	Photosynthesis [6]
NHB	LOC103853739	2	22908807	ubiquitin_carboxyl-terminal_hydrolase_21	

NHB	LOC103863123	4	2639421	Probable inactive receptor-like protein kinase At3g56050	
NHB	LOC103865205	4	17209360	cellulose_synthase-like_protein_B3%2C_transcript_variant_X2	Osmotic stress tolerance [9]
NHB	LOC103865206	4	17214041	NA	
NHB	LOC103865207	4	17220493	NA	
NHB	LOC117133654	4	17232983	receptor_like_protein_23-like	
NHB	LOC103865859	4	20185260	uncharacterized_methyltransferase_At2g41040%2C_chloroplastic	
NHB	LOC103867538	5	6115111	nucleolar_transcription_factor_1	
NHB	LOC103867544	5	6166731	uncharacterized_LOC103867544	
NHB	LOC103867553	5	6202720	myosin-10%2C_transcript_variant_X5	
NHB	LOC103835864	8	19823118	ubiquinone_biosynthesis_protein_COQ9%2C_mitochondrial	
NHB	LOC103847167	10	19384266	G-patch_domain-containing_protein_C1486.03%2C_transcript_variant_X2	
NHB	LOC103847185	10	19445556	uncharacterized_LOC103847185	

Supplementary References

1. Dorey, T., Schiestl, F. P. Plant phenotypic plasticity changes pollinator-mediated selection. Evolution **76** (12), 2930-2944 (2022).
2. Lee, S. C., & Luan, S. ABA signal transduction at the crossroad of biotic and abiotic stress responses. Plant, cell & environment, **35**(1), 53-60 (2012).
3. Pi, B., He, X., Ruan, Y., Jang, J. C., & Huang, Y. Genome-wide analysis and stress-responsive expression of CCCH zinc finger family genes in Brassica rapa. BMC Plant Biology, **18** (1), 1-15 (2018).

4. Rosado, A., Schapire, A. L., Bressan, R. A., Harfouche, A. L., Hasegawa, P. M., Valpuesta, V., & Botella, M. A. The Arabidopsis tetratricopeptide repeat-containing protein TTL1 is required for osmotic stress responses and abscisic acid sensitivity. Plant Physiology, **142** (3), 1113-1126 (2006).

5. Saha, G., Park, J. I., Jung, H. J., Ahmed, N. U., Kayum, M. A., Chung, M. Y., Hur, Y., Cho, Y. G., Watanabe, M., Nou I. S. Genome-wide identification and characterization of MADS-box family genes related to organ development and stress resistance in Brassica rapa. BMC genomics, **16** (1), 1-21 (2015).

6. Vassey, T. L., & Sharkey, T. D. Mild water stress of Phaseolus vulgaris plants leads to reduced starch synthesis and extracSupplementary Tableucrose phosphate synthase activity. Plant Physiology, **89** (4), 1066-1070 (1989).

7. Yang, F., Xu, F., Wang, X., Liao, Y., Chen, Q., & Meng, X. Characterization and functional analysis of a MADS-box transcription factor gene (GbMADS9) from Ginkgo biloba. Scientia Horticulturae, **212,** 104-114 (2016).

8. Zhang, J. H., Sun, H. L., Zhao, X. Y., & Liu, X. M. Arabidopsis Casein Kinase 1-Like 8 enhances NaCl tolerance, early flowering, and the expression of flowering-related genes. Journal of Plant Interactions, **11** (1), 138-145 (2016).

9. Zhu, J., Lee, B. H., Dellinger, M., Cui, X., Zhang, C., Wu, S., ... & Zhu, J. K. A cellulose synthase-like protein is required for osmotic stress tolerance in Arabidopsis. The Plant Journal, **63** (1), 128-140 (2010).
