# Supplementary material for: Biotic interactions promote local adaptation to soil in plants
Source: Nat Commun. 2024 Jun 18;15:5186. doi: 10.1038/s41467-024-49383-x (PMC11189560; doi:10.1038/s41467-024-49383-x)
Supplement: Supplementary file 4 — Description of Additional Supplementary Files [file 41467_2024_49383_MOESM4_ESM.docx]

**Description of Additional Supplementary Files**

**File Name**: Supplementary Data 1**. Dataset.**

**Description**: Traits values of plants growing in different soil (L = limestone, T= Tuff), and in different soil treatment (fertilizer: with use of fertilizer, nofertilizer: without use of fertilizer, standardized: grown in standardized soil, which provides optimal conditions for cultivation).

**File name**: Supplementary Data 2. **Dataset.**

**Description**: Phenotypic, attractiveness and reproductive traits among first and seven generation plants and those having evolved with or without aphid-herbivory, with or without bee-pollination and either in limestone or tuff soil. LHB: limestone line plants (L) growing with aphid-herbivory (H) and bee-pollination (B). LHH: limestone line plants (L) growing with aphid-herbivory (H) and hand-pollination (H). LNHB: limestone line plants (L) growing without herbivory (NH) and bee-pollination (B). LNHH: limestone line plants (L) growing without herbivory (NH) and hand-pollination (H). THB: tuff line plants (T) growing with aphid-herbivory (H) and bee-pollination (B). THH: tuff line plants (T) growing with aphid-herbivory (H) and hand-pollination (H). TNHB: tuff line plants (T) growing without herbivory (NH) and bee-pollination (B). TNHH: tuff line plants (T) growing without herbivory (NH) and hand-pollination (H).

**File name**: Supplementary Data 3. **R Script.**

**Description:** Script to produce the allele frequency change matrix.

**File name**: Supplementary Data 4. **R Script.**

**Description:** Script for calculating the breeding values for markers, and merge them with the AF change matrix; check for conditional neutrality and antagonistic pleiotropy pattern in AF change.

**File name**: Supplementary Data 5. **R Script.**

**Description**: Script to produce the list of annotate genes from the marker matrix.
